# Astroviruses in Rabbits

**DOI:** 10.3201/eid1712.110967

**Published:** 2011-12

**Authors:** Vito Martella, Paschalina Moschidou, Pierfrancesco Pinto, Cristiana Catella, Constantina Desario, Vittorio Larocca, Elena Circella, Krisztian Bànyai, Antonio Lavazza, Chiara Magistrali, Nicola Decaro, Canio Buonavoglia

**Affiliations:** University of Bari Aldo Moro, Valenzano, Italy (V. Martella, P. Moschidou, P. Pinto, C. Catella, C. Desario, V. Larocca, E. Circella, N. Decaro, C. Buonavoglia);; Hungarian Academy of Sciences, Budapest, Hungary (K. Bànyai);; Istituto Zooprofilattico Sperimentale di Lombardia ed Emilia Romagna, Brescia, Italy (A. Lavazza);; Istituto Zooprofilattico Sperimentale di Marche ed Umbria, Perugia, Italy (C. Magistrali)

**Keywords:** astrovirus, rabbit, enteritis, colitis, viruses

## Abstract

By screening rabbits with enterocolitis or enteritis complex and asymptomatic rabbits, we identified a novel astrovirus. The virus was distantly related (19.3%–23.7% aa identity) in the capsid precursor to other mammalian astroviruses within the *Mamastrovirus* genus**.** By using real-time reverse transcription PCR, with specific primers and probes and targeting a conserved stretch in open reading frame 1b, we found rabbit astrovirus in 10 (43%) of 23 samples from animals with enteric disease and in 25 (18%) of 139 samples from asymptomatic animals in Italy during 2005–2008. The mean and median titers in the positive animals were 10^2^× and 10^3^× greater, respectively, in the symptomatic animals than in the asymptomatic animals. These findings support the idea that rabbit astroviruses should be included in the diagnostic algorithm of rabbit enteric disease and animal experiments to increase information obtained about their epidemiology and potential pathogenic role.

## Abstract 2

A novel astrovirus was found more frequently in rabbits with enteric disease than in asymptomatic animals.

Astroviruses (AstVs) (family *Astroviridae*) are nonenveloped, and their genome is composed of a plus-sense single-stranded RNA of 6.4–7.3 kb, containing 3 open reading frames (ORFs) and a 3′ poly-A tail (*1*). Two ORFs, located at the 5′ end of the genome (ORF1a and ORF1b), encode nonstructural proteins, and ORF2, located at the 3′ end, encodes the capsid protein (*1*). AstVs were first identified by electron microscopy (EM) in 1975 in Scotland in fecal specimens of infants hospitalized with diarrhea (*2*). Subsequently, similar viruses were identified from several mammalian and avian species (*3**–**12*), including bats (*13*) and aquatic mammals (*14*). AstV infection is associated with gastroenteritis in most animal species and humans. AstVs are regarded as the second or third most common cause of viral diarrhea in children (*1*). Avian AstVs have also been associated with extraintestinal diseases, such as nephritis in chickens (*12*) and hepatitis in ducks (*11*). Even more notably, recently AstVs have been detected in the nervous tissues of minks with shaking disease (*15*) and in the central nervous system of a child with encephalitis (*16*). Also, novel human AstVs (MLB1, MLB2, VA1, HMO-C, HMO-B, HMO-A, VA-2) have been identified that are genetically unrelated to classical human AstVs (*17**–**19*) and more closely related to animal AstVs.

Rabbit enteritis, also referred to as enteritis complex (EC) or rabbit enterocolitis (REC), is a multiform enteric disease, characterized by a variety of symptoms. The syndrome can be caused by bacteria, viruses, and parasites. Moreover, environmental factors can alter rabbit physiology and impair rabbit welfare, thus increasing the effects EC/REC syndrome would have on rabbit production. Several different viruses have been isolated from rabbits with diarrhea, such as rotavirus, coronavirus, parvovirus, adenovirus, and caliciviruses (*20*). Whether natural outbreaks of enteritis can be caused by these viral agents alone or in conjunction/synergism with other pathogens is not clear, and the mechanisms of persistence/transmission are also not known.

Although AstVs have a peculiar star shape when purified fractions are observed in EM, which distinguishes them from other small, rounded viruses (SRVs), such as enteroviruses and caliciviruses, identifying them can be difficult when examining biologic samples because their typical morphologic features tend to be altered easily. During 1997–2005 surveillance by the National (Italian) Reference Centre for Viral Diseases of Rabbits, SRVs were identified by EM in 18 (3.49%) of 515 fecal samples from rabbits with enteric disease (*20*,*21*). In this study, we report the detection and characterization of AstVs in the intestinal contents of rabbits affected by EC/REC.

## Materials and Methods

### Samples from Animals with Enteritis (Collection A)

A total of 23 pooled (2–5 animals) and single samples (various tracts of small and large intestine and/or intestinal contents of rabbits with enteritis) were collected from 23 commercial rabbitries in Italy during 2005–2008. EC/REC of various degrees of severity was described in the herds, with animals ranging in age from 35 to 55 days (Table 1). The samples were sent to the laboratories of the Istituto Zooprofilattico Sperimentale della Lombardia e dell’Emilia Romagna, Brescia, Italy. After routine laboratory investigations (bacteriologic and parasitologic analysis for enteric pathogens), the samples were stored at −80°C. For bacteriologic analysis, samples were inoculated on MacConkey and Columbia blood agar (Liofilchem, Teramo, Italy), under aerobic and anaerobic conditions, at 37°C for 48 h. The presence of parasites was investigated by microscopy observation of smears made from the fecal or intestinal content specimens, both directly and after concentration by flotation.

**Table 1:** Astrovirus-positive samples from commercial rabbitries with enteric diseases and results of electron microscopy, clinical observations, and pathologic, bacteriologic, and parasitologic investigations, Italy, 2008*

Sample no.	Place of origin	GE/mL feces	Electron microscopy results†	Clinical observations	Pathologic findings	Bacteriologic findings (intestinal tract)	Parasitologic findings (intestinal tract)
1	Pavia	3.1 × 10^8^	Rotavirus ++ Coronavirus +++	Age 48 d, enteric syndrome	Enterocolitis	*Escherichia coli, Clostridium perfrigens*	Negative
2	Brescia	8.5 × 10^3^	Negative	Age 35 d, enteric syndrome	Enterocolitis	*E. coli*	Negative
3	Brescia	1.5 × 10^6^	Negative	Age 55 d, enteric syndrome; fatal in 24 h	Enteritis with tracts containing fluids and tract filled with feces	Negative	*Coccidia*
4	Nuoro	1.0 × 10^8^	Rotavirus +/–	NA	Catarral enteritis	*E. coli*	*Coccidia*
5	Brescia	9.7 × 10^7^	Rotavirus ++ Phages ++	Age 51 d, enteric syndrome, high mortality	Enterocolitis with swollen colon	*E. coli*	*Coccidia*
6	Brescia	1.0 × 10^9^	Coronavirus+	Age 51 d, enteric syndrome, high mortality	Enteritis	*E. coli*	*Coccidia*
7	Brescia	3.8 × 10^7^	Rotavirus +++ Phages +++	Age 51 d, enteric syndrome	Enterocolitis, with swollen colon	*E. coli*	Negative
8	Padova	7.3 × 10^3^	Rotavirus +++	NA	Catarral enteritis	*E. coli*	Negative
9	Lecco	1.4 × 10^10^	Rotavirus +++	NA	Typhlitis and colitis, swollen tracts of the gut with fluid content	*E. coli* (gut), *Streptococcus* spp. (liver and spleen)	*Coccidia*
10	Cagliari	6.5 × 10^7^	Negative	NA	Catharral enteritis	*E. coli*	*Coccidia*

*GE, genome equivalent; NA, not available. †+/–, very low positivity; +, low positivity; ++, discrete positivity; +++, strong positivity.

### Samples from Asymptomatic Animals (Collection B)

A total of 139 fecal samples were collected from postweaning rabbits (30–35 days of age) from 15 herds. EC/REC disease was not reported in the history of the herds, and the animals were overtly healthy at the time of sampling. The samples were stored at −80°C until use.

### RNA Extraction and Screening for AstVs by Reverse Transcription PCR

RNA extracts were prepared from 10% homogenates in phosphate-buffered saline, pH 7.3, after clarification by centrifugation at 10,000 × *g* for 1 min. Viral RNA was extracted by using the QIAamp viral RNA kit (QIAGEN GmbH, Hilden, Germany). The samples from collection A were used for an initial screening with a broadly reactive primer pair, targeted to the ORF1b region of AstV (*13*). The initial screening showed PCR amplicons of the expected size (409 bp). Sequence analysis indicated that the sequences displayed 91.9%–96.6% nt identity to each other. BLAST (www.ncbi.nlm.nih.gov) and FASTA (www.ebi.ac.uk/fasta33) with default values were used to find homologous hits in the sequence databases. The sequences displayed the highest identity (69.0%–71.3% nt) to an AstV from a California sea lion (GenBank accession no. FJ890353), thus confirming AstV infection.

### Real-Time Quantitative PCR for Rabbit AstVs

The partial ORF1b sequences generated with the AstV broadly reactive ORF1b primers were used to design more specific primer sets and probes for reverse transcription PCR and RT real-time quantitative PCR (RT-qPCR) able to identify and quantify the rabbit AstVs (Table 2). Primers and TaqMan probes were designed by using Beacon Design software version 2.0 (Premier Biosoft International, Palo Alto, CA, USA). The RT-qPCR was performed by using a 2-step protocol and a real-time thermocycler (i-Cycler iQTM Real-Time Detection, Bio-Rad Laboratories, Hercules, CA, USA). The ORF1b amplicon (409 bp) of the rabbit AstV strain Nausika/08/ITA was cloned into pCR4-TOPO vector (TOPO TA cloning, Invitrogen, Milan, Italy) and transcribed in vitro with Ribo-MAXTM Large Scale RNA Production System-T7 (Promega Italia, Milan, Italy) from the T7 promoter, according to the manufacturer’s guidelines. The transcribed RNA was quantified and used to generate an RNA standard curve. The detection limit was 10 genomic equivalents (GEs)/50 μL-reaction (cycle threshold = 42.67), corresponding to 3.6 × 10^2^ GE/g of fecal sample. No other enteric viruses, including rabbit rotaviruses and human, canine, porcine, and avian AstV strains, were detected. This RT-qPCR is sensitive and specific for the detection of rabbit AstV.

**Table 2:** Primers used for detection and sequencing analysis of rabbit astroviruses, Italy, 2008

Primer	Sequence, 5′ → 3′	Sense	Reference
702VM-Pb	6FAM-TCTCAACAGGTATGTCGTCCTCCCTTCTGG-BHQ1	+	This study
683VM-F	CCATATAYAAGTGGTATTGCAARCA	+	This study
684VM-R	TTCCGCTGRATGGTRACCTC	–	This study
panAstVFor1	GARTTYGATTGGRCKCGKTAYGA	+	(*13*)
panAstVFor2	GARTTYGATTGGRCKAGGTAYGA	+	(*13*)
panAstVRev	GGYTTKACCCACATNCCRAA	–	(*13*)
VN3T20	GAGTGACCGCGGCCGCT_20_	–	(*23*)

### EM Observation

The samples that contained AstV RNA were processed for EM observation (*22*). Briefly, the feces were diluted 1:10 in distilled water, vortexed, and centrifuged for 20 min at 4,000 × *g* and again for 10 min at 9.300 × *g* for clarification. The supernatant was then ultracentrifuged (Beckman Airfuge, Fullerton, CA, USA) for 15 min at 82.000 × *g*. After negative staining with 2% sodium phosphotungstate (pH 6.8), samples were examined by using a Philips CM10 electron microscope.

### Molecular Characterization of Rabbit AstV Strain Nausika/08/ITA

To determine the sequence and genomic organization of the novel rabbit AstV, we selected a sample containing 1.3 × 10^10^ GE/*g* fecal sample (strain Nausika/ITA/08). A 3.4-kb region at the 3′ end of the genome was amplified by RT-PCR as described by Wang et al. (*23*). cDNA was synthesized by SuperScript III First-Strand cDNA synthesis kit (Invitrogen Ltd, Paisley, UK) with primer VN3T20. PCR was then performed with TaKaRa La Taq polymerase (TaKaRa Bio Europe SAS, Saint-Germain-en-Laye, France) with forward primer and VN3T20. Finally, the amplicon was purified and cloned by using TOPO XL Cloning Kit (Invitrogen Ltd). Additional primers also were designed to determine the complete 3.4-kb sequence by an overlapping strategy. The sequence was deposited in GenBank under accession no. JN052023.

Sequence editing and multiple alignments were performed with Bioedit software package version 2.1 (*24*). Phylogenetic analysis (neighbor-joining and unweighted pair group method) with arithmetic mean with bootstrap analysis (1,000 replicates) and no-distance correction was conducted by using MEGA4 software (*25*).

### Analysis of RNA-Dependent RNA Polymerase (ORF1b) and Capsid Protein (ORF2) of Strain Nausika/08/ITA

Pair-wise identity in the ORF1b and full-length capsid protein of strain Nausika/08/ITA to a selection of AstV strains was determined by using multiple alignments generated with Bioedit software package version 2.1 (*24*). The values were calculated with the uncorrected distance method by using amino acid sequence alignment without removing the gaps, including sequences of human and animal AstVs. The strain and sequences used are listed in Table 3.

**Table 3:** Comparison of full-length capsid protein of strain rabbit/Nausika/08/ITA and that of various mammalian and avian astroviruses*

GenBank accession no./ species/strain	% aa identity to rabbit/Nausika/08/ITA†	
	RdRp (ORF1b)	Capsid (ORF2)
AY720892/human/AstV1	62.7	21.8
L06802/human/AstV2	–	22.3
DQ630763/human/AstV3	–	22.5
DQ070852/human/AstV4	–	22.2
U15136/human/AstV5	–	22.0
GQ495608/human/AstV6	61.4	21.9
AF248738/human/AstV7	52.8	22.8
AF260508/human/AstV8	61.4	22.7
FJ973620/human/VA1	–	20.1
GQ502193/human/VA2	52.5	19.7
FJ222451/human/MLB1	61.4	19.6
AF056197/cat	–	21.7
Y15937/sheep	52.9	20.5
AY179509/mink	54.1	20.0
AB037272/pig	–	23.5
FJ890351/CSL/AstV1	52.1	19.3
FJ890352/CSL/AstV2	60.6	23.3
FJ890355/bottlenose dolphin	61.4	23.7
EU847155/bat/AstV1	–	22.8
FJ57174/bat/LC03	–	22.7
FJ57065/bat/LD38	50.9	22.3
HM045005/dog/Bari/08	57.7	23.7
HM450382/rat/RS126/HKG/07	57.7	20.6
GU985458/mink/SMS-AstV/Swe/01	54.1	19.6
AB033998/chicken/ANV-1	36.8	14.9
AB046864/chicken/ANV-2	36.4	14.5
Y15936/turkey/AstV1	36.8	15.5
AF206663/turkey/AstV2	35.6	14.2
FJ434664/duck/C-NGB/China/08	37.2	13.4

*RdRP, RNA-dependent RNA polymerase; ORF, open reading frame; AstV, astrovirus; –, values not calculated; CSL, California sea lion; ANV, avian nephritis virus. †Full-length aa capsid sequence and partial (245-aa residues) RdRp at the at the C-terminus were used to calculate identities.

## Results

### Screening of Samples from Collections A and B for rabbit AstV by RT-qPCR

Rabbit AstV was detected by RT-qPCR in 10 (43.49%) of 23 samples from collection A **(**Table 1) and in 25 (17.98%) of 139 from collection B. Rabbit AstV was detected in 12 (80%) of the 15 rabbit herds sampled, with the herd prevalence ranging from 9% to 50%. Virus titers (GE/μL RNA extract) in collection A ranged from 2.0 × 10^1^ to 3.8 × 10^7^ (mean value 4.3 × 10^6^, median value 2.1 × 10^5^), and in collection B from 1.2 × 10^1^ to 1.7 × 10^6^ (mean value 7.6 × 10^4^, median value 1.5 × 10^2^). By comparing the 2 groups using Software R version 2.8.1 (www.r-project.org), by the χ^2^ test, the positivity rates differed significantly between groups A and B (p = 0.0132; p<0.05). To assess whether a significant difference in virus shedding (titers) between symptomatic and asymptomatic animals, the virus titers of group A and B were compared by the nonparametric Mann-Whitney U test. In this analysis, the 2 animal groups differed significantly (p = 0.0137; p<0.05).

### EM Observation

Upon EM observation, none of the AstV-positive samples contained SRV-like particles. Rotaviruses, coronaviruses, and phages were detected in 8 samples in various combinations, and 2 samples did not contain viral particles (Table 1).

### Molecular Characterization of Rabbit AstV Strain Nausika/08/ITA

A 3.4-kb (3,395 nt) sequence at the 3′ end of the genome of strain Nausika/08/ITA was determined. The sequence spanned the 3′ end of ORF1b, the full-length ORF2 and the 3′ noncoding region (NCR) to the poly-A tail. The 3′ end of ORF1b comprised 759 nt, encoding for a 252-aa polypeptide fragment at the C-terminus of the RdRp. By pair-wise comparison in the partial RdRp, the highest aa identity (62.7%) was to human AstV type 1 (GenBank accession no. AY720892). Identity to other mammalian AstVs ranged from 50.9% to 61.4% aa, whereas identity to avian AstVs ranged from 31.0% to 37.5% aa.

An 8-nt overlap occurred between the termination codon of ORF1b and the initiation codon of ORF2. The highly conserved nt stretch upstream of ORF2, ATTTGGAGNGGNGGACCNAAN_5–8_ATGNC, which is believed to be part of a promoter region for synthesis of subgenomic RNA (*26*), was nearly completely conserved in the sequence of strain Nausika/08/ITA. The ORF2 was 2,559 nt and encoded for a capsid protein of 852 aa, with a predicted molecular mass of 84.7 kDa. The NCR was 85 nt. By pair-wise comparison, the highest identity (23.7%) in the capsid protein was found to a canine AstV, strain Dog/Italy05 and to a bottlenose dolphin AstV. Identity to other mammalian AstVs ranged from 19.3% to 23.5% aa, whereas identity to avian AstVs ranged from 13.4% to 14.9% aa (Table 2). The highly conserved motive SRGHAE at the C-terminus of VP1 was not present (*27*). By phylogenetic analysis, the strain was found to segregate in the *Mamastrovirus* genogroup (Figure).

**Figure:**
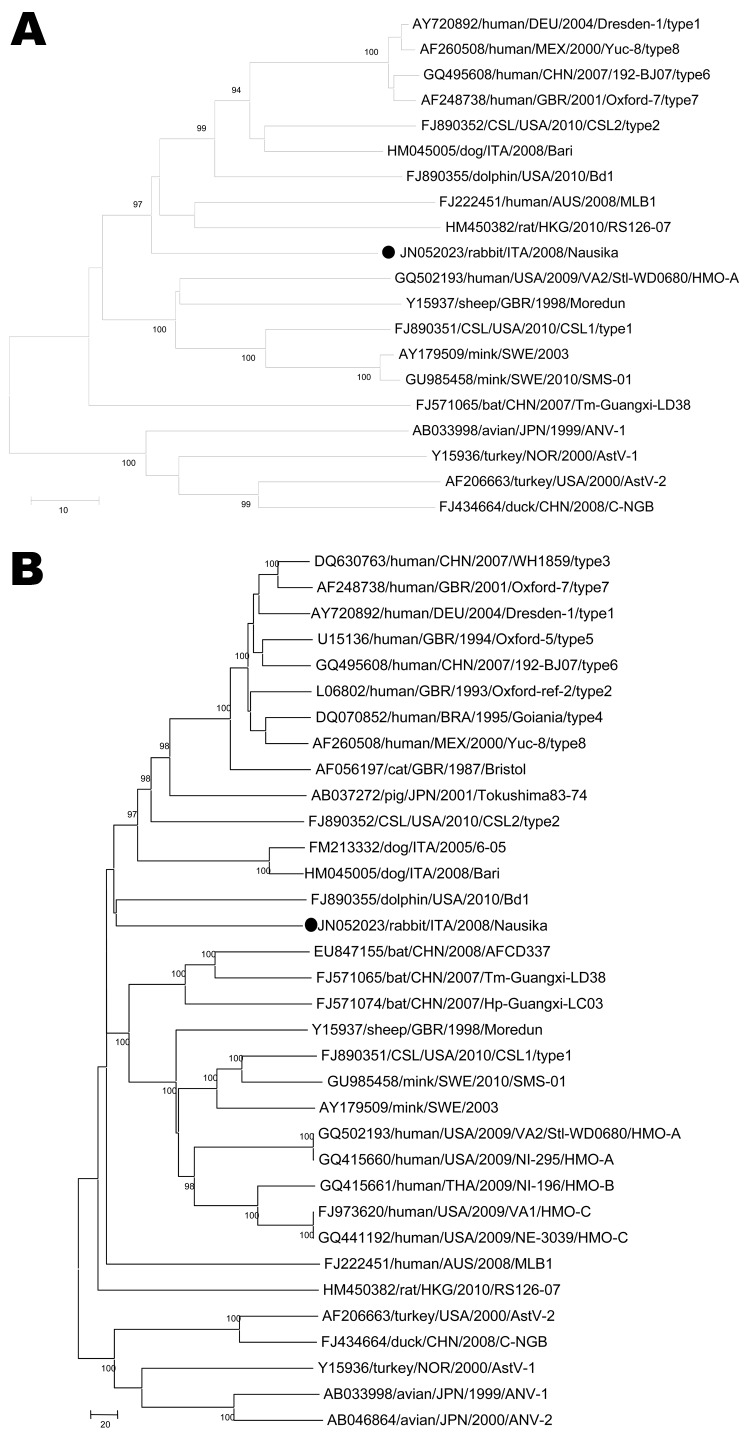
Phylogenetic trees constructed on the partial (245 aa) RNA-dependent RNA polymerase (panel A, RdRp) (open reading frame [ORF] 1b) and the full-length capsid precursor (panel B, ORF2) amino acid sequences. Black circles indicate strain identified in this study. The trees were constructed by using a selection of astrovirus (AstV) strains. Country names are abbreviated. Scale bars indicate the number of amino acid substitutions per 100 residues. Bootstrap values <90% are not shown. CSL, California sea lion; ANV, avian nephritis virus.

With some exception, most AstVs have a conserved RNA secondary structure, referred to as the stem-loop II-like motif (s2m), located at the 3′ end of the genome in the 3′ NCR (*28*). Nucleotide alignment of the 150 nt at the 3′ terminus of the rabbit AstV Nausika/08/ITA genome and other viruses known to contain the stem-loop motif suggested that Nausika/08/ITA does not have this conserved nucleotide motif (data not shown).

## Discussion

Rabbit EC/REC syndrome is a multiform enteric disease characterized by a variety of symptoms. The syndrome may be multifactorial with several microorganisms acting in synergy and with environmental factors also altering or influencing rabbit physiology, metabolism, and immune response. Within this miscellaneous group of enteric pathologies, some diseases appear to possess peculiar features, such as the epizootic rabbit enteropathy, enterotoxemia caused by *Clostridium spiriforme* and *C. perfrigens* (*29*), *C. difficile* (*30*) infection, Tyzzer disease (caused by *C. piliformis)*, mucoid enteropathy, *Escherichia coli* enteritis, and coccidiosis (*31*).

Several viruses have been identified from rabbits with diarrhea (*20*), but whether viruses can act as primary agent of enteritis is not clear. Experimental infection of rabbits with rotavirus has shown that the rotavirus-induced disease is age restricted to the neonatal period (<2 weeks), although natural infection has been associated with disease in animals after weaning (28–45 days of age) (*32*). Also, maternally derived immunity protects young rabbits up to 2 months of age and may influence the evolution of virus infection or disease. Accordingly, much additional work remains to elucidate the viral pathogenicity and immunology of most enteric viruses of rabbits.

By EM observation, SRV-like viral particles have been seen sporadically in rabbits with EC/REC disease (*20**,**21*), but the exact nature of the observed SRVs, was not investigated. By using an AstV broadly reactive set of primers, we could detect AstV RNA in the intestinal contents of rabbits affected by EC/REC syndrome and the sequences obtained were used to generate more specific diagnostic tools. By rescreening the samples (collection A) with a RT-qPCR, AstV RNA was detected in 43.49% (10/23) of the samples tested. The mean titer in the AstV-positive samples from collection A was 4.3 × 10^6^ GE/μL RNA extract, corresponding to ≈1.5 × 10^9^ GE/mL feces. Notably, by EM observation, none of the AstV-positive samples was clearly found to contain SRV-like particles (Table 1). Immune EM that uses specific hyperimmune or convalescent-phase serum specimens could be necessary to improve the sensitivity of the EM technique. Also enzyme- or pH-mediated alterations in virus morphologic features could be triggered during conservation of samples and therefore hamper recognition of these SRVs. Overall, rabbit AstVs can be assumed to be easily undetected in EM-based surveys, thus leading to underestimation of the potential role of SRVs in rabbit EC/REC syndrome.

To better assess the epidemiology of these viruses, we analyzed a collection of samples obtained from asymptomatic animals (at 30–35 days of age). In these samples, rabbit AstV RNA was detected in 17.98% (25/139) of the samples from 12 of 15 herds. The mean titer in samples from collection B was 7.6 × 10^4^ GE/μL RNA extract, corresponding to 2.7× 10^7^ GE/mL feces, and this value (mean) was ≈10^2^ times lower than in the samples from collection A. Accordingly, the prevalence rates and the virus shedding titers differed markedly and significantly between the 2 sample groups.

The rate of detection of enteric viruses (noroviruses) in humans is significantly higher for symptomatic (37.2%) than asymptomatic patients (14.1%) (*33*). Also, increased viral load in the feces has been associated with greater severity of gastroenteric disease in children infected by group A rotavirus (*34*) and with longer duration of diarrhea, but not with greater disease severity, in children infected with AstVs (*35*). However, the differences observed in rabbit AstV prevalence, and titers between the 2 sample collections are not necessarily suggestive of a pathogenic attitude or role for rabbit AstVs and must be interpreted with caution. Bias in AstV distribution could be accounted for by the sampling inclusion criteria (age of group B animals) or by the relatively small number of samples analyzed.

Regardless, the samples from collection A and B were from herds of different regions in Italy (Emilia Romagna, Lombardia, Sardegna, Umbria, and Veneto). Accordingly, our findings suggest that rabbit AstVs are common in rabbit herds. Cycles of infection in newly susceptible animals, coupled with stability/resistance of SRVs and management conditions (high density of animals), could account for the high prevalence rates observed.

Upon sequence analysis of the full-length capsid sequence, the rabbit strain Nausika/08/ITA was distantly related (19.3%–23.7%-aa identity) to other mammalian AstVs. Similar ranges of genetic diversity/heterogeneity in this portion of AstV genome (ORF2) have been observed in other AstVs found in mammals (*36*). Notably, the 5′ end of ORF2 appeared to be more conserved than the central region and the 3′ end. AstVs contain a conserved region at the junction between ORF1b and ORF2 (*26*). The exact role of this sequence is not known, but it may be a regulatory element of the subgenomic RNA that encodes for ORF2. This region appeared nearly completely conserved in the sequence of strain Nausika/08/ITA. Unlike the situation with other mammalian AstVs, but not unusually, the highly conserved motif SRGHAE at the C-terminus of the capsid protein (*27*) was not found. In addition, the stem-loop II-like motif (s2m) at the 3′ end of the genome in the 3′ nontranslated region (*28*) was not present in strain Nausika/08/ITA. This motif is present in most AstVs, with the exception of turkey AstV 2, human AstV MLB1, and rat AstV (*36**,**37*). This motif is also found in some coronaviruses and equine rhinovirus serotype 2. The conservation of such a sequence motif across multiple viral families suggests that it may play a broad role in the biology of positive-stranded RNA viruses (*28*), although the exact function of this conserved region is still unknown.

In conclusion, we identified a novel AstV in rabbits. Also, we developed an RT-qPCR useful for detection and quantification of rabbit AstV and gathered evidence that AstVs are common in the intestinal content/feces of both symptomatic and asymptomatic rabbits. Including rabbit AstV in the diagnostic algorithms of rabbit enteritis and animal experiments will be useful in clarifying whether these enteric viruses play a role in rabbit EC/REC syndrome.
